# Microneedle Array Electrode-Based Wearable EMG System for Detection of Driver Drowsiness through Steering Wheel Grip

**DOI:** 10.3390/s21155091

**Published:** 2021-07-27

**Authors:** Afraiz Tariq Satti, Jiyoun Kim, Eunsurk Yi, Hwi-young Cho, Sungbo Cho

**Affiliations:** 1 Department of Electronics Engineering, Gachon University, Seongnam 13210, Korea; afraiztariq@gmail.com; 2 Department of Exercise Rehabilitation & Welfare, Gachon University, Incheon 21936, Korea; eve14jiyoun@gachon.ac.kr (J.K.); yies@gachon.ac.kr (E.Y.); 3 Department of Physical Therapy, Gachon University, Incheon 21936, Korea; hwiyoung@gachon.ac.kr; 4 Department of Health Science and Technology, GAIHST, Gachon University, Incheon 21999, Korea

**Keywords:** driver drowsiness, microneedle electrode, EMG, STFT

## Abstract

Driver drowsiness is a major cause of fatal accidents throughout the world. Recently, some studies have investigated steering wheel grip force-based alternative methods for detecting driver drowsiness. In this study, a driver drowsiness detection system was developed by investigating the electromyography (EMG) signal of the muscles involved in steering wheel grip during driving. The EMG signal was measured from the forearm position of the driver during a one-hour interactive driving task. Additionally, the participant’s drowsiness level was also measured to investigate the relationship between muscle activity and driver’s drowsiness level. Frequency domain analysis was performed using the short-time Fourier transform (STFT) and spectrogram to assess the frequency response of the resultant signal. An EMG signal magnitude-based driver drowsiness detection and alertness algorithm is also proposed. The algorithm detects weak muscle activity by detecting the fall in EMG signal magnitude due to an increase in driver drowsiness. The previously presented microneedle electrode (MNE) was used to acquire the EMG signal and compared with the signal obtained using silver-silver chloride (Ag/AgCl) wet electrodes. The results indicated that during the driving task, participants’ drowsiness level increased while the activity of the muscles involved in steering wheel grip decreased concurrently over time. Frequency domain analysis showed that the frequency components shifted from the high to low-frequency spectrum during the one-hour driving task. The proposed algorithm showed good performance for the detection of low muscle activity in real time. MNE showed highly comparable results with dry Ag/AgCl electrodes, which confirm its use for EMG signal monitoring. The overall results indicate that the presented method has good potential to be used as a driver’s drowsiness detection and alertness system.

## 1. Introduction

Drowsiness is defined as a natural tendency to fall asleep. The transition from awake to asleep is recognized as sleep onset (SO) [1], commonly known as drowsiness. Driver drowsiness results in over 20% of road accidents [2,3], reported as one of the leading causes of road fatalities. The most common factors associated with driver drowsiness are sleep deprivation, duration of driving, monotonous environments, drug and alcohol use, and chronic sleepiness [4]. Another factor associated with a road accident is risky driving behaviors by young drivers. Watling studied the behavior of young drivers who continue to drive while drowsy [5]. It was reported that a large number of drivers (70–73%) choose to drive even though they were aware of their increased level of drowsiness.

Over the past few decades, researchers have investigated several methods to detect driver drowsiness at an early stage and its prevention. Most of the researchers used behavioral, vehicle-based, and physiological measurements to assess driver drowsiness. Behavioral measures mostly employ head pose, eye closure, yawning, and facial expression [6]. There are some disadvantages associated with these noninvasive measures; environmental factors such as brightness, illumination, and road conditions affect the accuracy of the measurement [7]. On the other hand, some researchers implemented the vehicle moment and steering wheel moment method to detect driver drowsiness. However, vehicle moment-based driver drowsiness detection is limited because the values are easily affected by external factors such as weather conditions and the geometric characteristics of roads [8]. Previously, some researchers have reported the detection of driver fatigue by monitoring steering wheel grip force [9,10]. Steering wheel grip force-based driver fatigue detection has several advantages over other measures. The measurement can be used in both nighttime and daytime, and it is not influenced by the external environments and illumination [11]. According to Polychronopoulos et al. (2004), the steering wheel grip force is a good option to be used along with other measures like eye movement [12]. Lin et al. (2007) studied a grip force-based multi-sensor driver fatigue detection system [13]. However, most of the previous research used the force-sensing resistor (FSR) on the steering wheel to measure grip force during driving. Among all methods, physiological measurement has better reliability and accuracy [14]. Physiological features play an important role in fatigue recognition because people have limited control over them, making them a reliable source of information on a person’s emotions [15]. Two of the most significant observable features such as the brain activity from an electroencephalogram (EEG) and muscle activity features from an EMG were used in previous studies to detect driver drowsiness. The EEG is an important method to be used for driver drowsiness detection, as it provides direct information about human cognitive states [16]. The physiological features related to muscle activity have the advantage in that they can be measured using a very small wearable measurement system and electrodes in comparison to an EEG, which requires a large number of electrodes and a large cap [17].

During driving, the transition from alertness to drowsiness causes a change in physiological features. A study by Chowdhury et al. reported a reduction in EMG signal amplitude due to an increase in drowsiness [18]. The study conducted by Dong-Mei et al. reported that an increase in handgrip force increases associated muscle signal amplitude and vice versa [19]. Muscle activity from the steering wheel grip could be investigated for driver drowsiness detection. The electrical activity associated with muscle contraction or relaxation can be recorded by the EMG technique [20].

**Figure 1:**
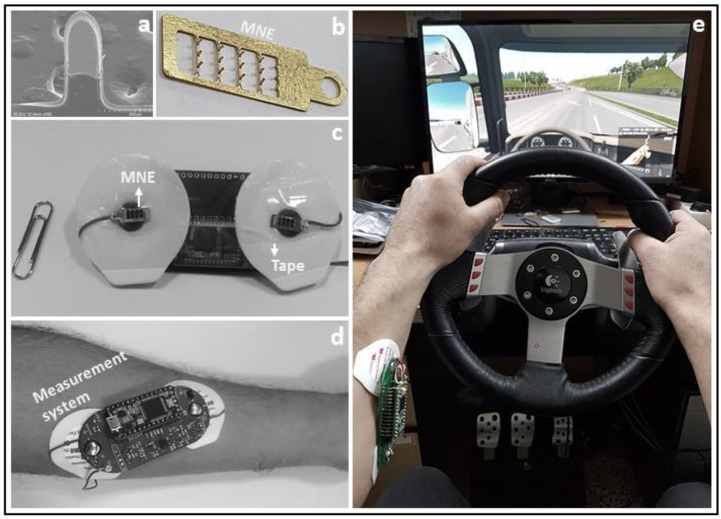
(**a**) SEM image of a single microneedle; (**b**) MNE; (**c**) MNE attached with adhesive dressing for skin contact; (**d**) Measurement system with MNE placed on forearm position; (**e**) Driving simulation system with display.

This study investigates the relationship between a driver’s drowsiness level and muscle activity associated with steering wheel grip during simulated driving. The muscle activity signal was acquired from the forearm position of participants while they were involved in interactive driving. A study conducted by Sidek et al. reported that the flexor digitorum superficialis (FDS) and extensor digitorum superficialis (EDS) contribute to handgrip force [21]. The EMG electrodes were placed on these muscles to investigate the electrical activity of these muscles associate with driver’s steering wheel grip. Time-domain and frequency domain analysis was used to examine the time and frequency response of the resultant EMG signals. STFT and spectrogram were used to assess the resultant signals’ frequency distribution over time [22]. Additionally, an amplitude shift-based driver drowsiness detection algorithm is proposed to detect and alert the driver in real time. Considering the recent trend in MNE-based wearable technology, we investigated the previously presented MNE shown in Figure 1b to acquire an EMG signal during the driving simulation task [23]. The signal acquired with minimally invasive MNE is highly comparable with the one obtained with a wet Ag/AgCl electrode, which shows MNE promise for quality EMG signal monitoring. The results show that the presented method and the proposed algorithm have good potential to be used for driver drowsiness early detection and prevention studies. For future studies, other physiological signals such as an electroencephalogram (EEG) or electrooculogram (EOG) could be used along with EMG to develop a better understanding of driver drowsiness stages. As the present study is related to the physiological changes that occur during drowsiness state, thus, after fusing the further physiological features, this study could be investigated for the sleep stage analysis used in sleep disorder studies [24].

## 2. Materials and Methods

### 2.1. Materials

A driving simulation system from Logitech (International S.A.) was used to set the driving environment, as shown in Figure 1c. MNE from a previous study [23] was used to acquire the EMG signal from the skin by a minimally invasive procedure. Ag/AgCl monitoring electrodes from 3M Health Care (Seoul, Korea) were also used. A LabVIEW-based graphical user interface was developed for signal visualization on PC. All tests were performed in normal conditions and were repeated at least three times. Written consent was obtained from volunteers before the experiment, and all the ethics were followed during the examination.

### 2.2. AD8232-Based EMG Measurement System

To measure EMG, a previously presented AD8232 integrated circuit-based bio measurement device (Analog Devices, Inc., Norwood, MA, USA) was used after some component modifications to increase the frequency band for EMG signal monitoring [23]. Since the EMG signal is very low in amplitude, it needed to be amplified before any digitization. To avoid the amplification of unwanted noisy signals, high-pass filtering was conducted in the early stages. It is critical to apply low-pass filtering on the analog signal before digitization to avoid an anti-aliasing effect. The AD8232, which was used in the present study, is a tiny packaged integrated circuit that can extract, filter, and amplify small biopotential signals in the analog domain and assist in real-time biosignal monitoring [25]. The special instrumentational amplifier presented in AD8232 allows for high-pass filtering and gain in one stage. Externally, an RC network is connected to this amplifier to set the high-pass filter’s frequency. Studies have reported that low-frequency noise, such as movement artifacts, occurs significantly in the range of 0–20 Hz [26,27]. To avoid low-frequency noises, the high-pass filter’s cutoff frequency was set to 20 Hz.

**Figure 2:**
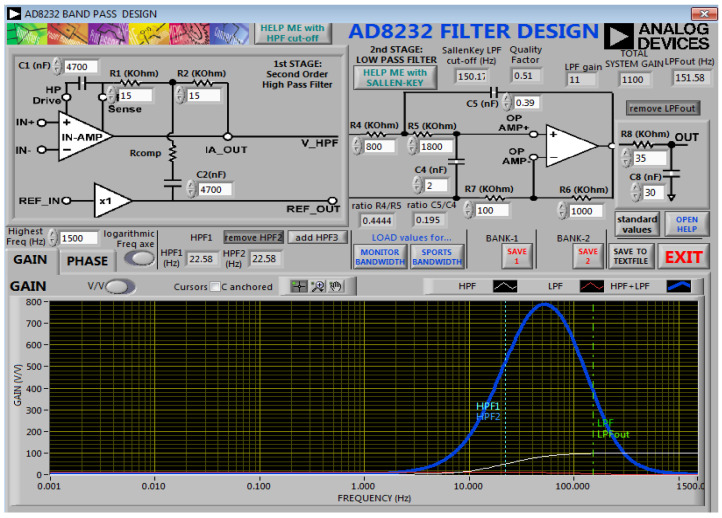
Component selection and response for the band-pass filter using Filter Design software [28]. Copyright © 2019, Analog Devices, Inc. All Rights Reserved.

To select the component values for the selected cutoff frequency range, the AD8232 filter design tool provided by Analog Devices was used, as shown in Figure 2. The software provides filter implementation for predesigned applications. Additionally, the software displays the representation of bandwidth and responses for the externally connected circuit to AD8232.

**Figure 3:**
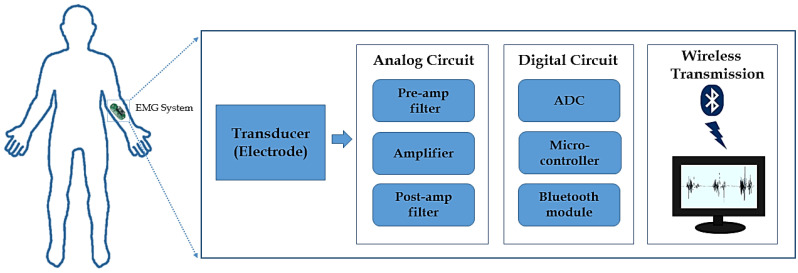
Schematic illustration of the EMG measurement system.

The AD8232 also has an operational amplifier that creates a three-pole low-pass filter to remove high-frequency noise. The cutoff frequency of the low-pass filter was set by changing the component associate with op-amp A1 presented in AD8232. The high cutoff frequency used in the low-pass filter was set to 150 Hz for EMG measurement. According to the Nyquist criterion, the signal must be sampled at least twice the highest frequency in the signal. Thus, we used a sampling frequency of 300 Hz. The EMG measurement system is less disturbing to the driver because of its small size. The schematic illustration of the EMG system is shown in Figure 3.

### 2.3. Driver Drowsiness Detection System Design and Procedure

All participants were asked to sleep less than 5 h the night before the experiment to induce drowsiness during the experiment. Before the start of the experiment, the participants were briefly introduced to the experimental procedure and study purpose. The participants were allowed for 15 min before the experiment to understand the entire setup and operate the system. The room temperature was maintained at 24–26 °C, and lighting conditions were constant throughout the experiment. The driving simulation was operated for 1 h by each participant. A 50 km closed-loop highway was selected for the driving, and the driving speed was 60 km/h. A quasi-circular loop with no sharp turns was chosen to prevent disturbing drowsy drivers. The curvature radius was 15 km; thus, if a driver does not control the steering wheel, the car may exit the road. The route was the same for all drivers. The drivers were asked to keep the steering wheel grip and ensure controlled turning. Previous studies showed that the most number of road accidents were recorded during the peak drowsiness periods 2:00 p.m. to 4:00 p.m. and 2:00 a.m. to 6:00 a.m. [29]. For the present study, the participants were asked to drive from 3:00 p.m. to 4:00 p.m.

Three MNEs were used to acquire the EMG signal. MNE electrodes were attached with a wet sticky dressing, and the conductive part of the wet dressing was covered with tape. Thus, only MNEs were used to obtain the signal from the skin, as shown in Figure 1c. The electrodes were placed on the forearm position of subjects and were attached to the developed EMG monitoring system, as shown in Figure 1d. MNE’s were sterilized using 70% ethanol before applying to the skin. The two working MNEs for IN+ and IN- were placed on the flexor carpi radialis and flexor carpi ulnaris, respectively. It was observed that if the placement of the IN+ and IN- electrodes is reversed, the output signal shape is also inverted. The reference MNE was placed on the backside of the lower arm of the extensor digitorum muscle. The position of the reference electrode can be changed and selected freely, but the reference electrode should not be placed on the same muscle as the investigated muscle used for IN+ and IN- electrode placement, which will decrease noise rejection [30]. The MNE position on the forearm is shown in Figure 1a. The same electrode placement position, same driving route, and other instructions provided to drivers were used for all experiments.

### 2.4. EMG Test Comparison of MNE vs. Ag/AgCl Electrodes

Before the driver drowsiness test with MNE, it was crucial to examine the biosignal acquiring capabilities of MNE. The EMG signal was acquired with the MNE and compared with the signal obtained using Ag/AgCl electrode. The examination study of MNE signal acquiring capability does not require a full simulated driving task. Therefore, the muscle signal generated during strong and weak handgrip was attained while the subject was not driving but holding the steering wheel. The same electrode placement positions were selected for both MNE and Ag/AgCl electrodes, as discussed in the previous section. After the electrodes were placed on the forearm, the candidate was asked to hold the steering wheel first with a strong grip and then with a weak grip and to repeat the same three times. Only one participant was tested for this study. The data were recorded and sent to the PC. Real-time data plotting along with the file saving system option based on the developed GUI was used to visualize and record the signal. After this MNE signal quality test, it was confirmed that the MNE could be used for other experiments.

### 2.5. Driver Drowsiness vs. Muscle Activity Test

The driver’s muscle activity signal associated with steering wheel grip was examined. Three participants were asked to drive the simulated driving system for 1 h. The EMG signal was acquired from the forearm position of the subjects. Three MNEs were used to acquire the targeted EMG signal. The same electrode placement positions and other conditions discussed in Section 2.3 were used for this test. While the subjects performed the driving task, the EMG signal was acquired; after the signal conditioning in the early stages, the signal was digitized by the microcontroller. The digital signal was directed to the PC over Bluetooth.

To validate the measurement, the drowsiness of the subjects was also measured during driving. Subjective drowsiness was measured on a degree of a 7-point scale. After every 10 min, the subjects were verbally asked about the degree of drowsiness. They were told to report a number between 1 and 7 (level 1, not drowsy at all; level 7, highly drowsy). The experiment was repeated with three healthy volunteers, and three readings were taken for each participant. The average and standard errors were calculated.

### 2.6. Frequency Domain Analysis of the Signal

The time-frequency representation (TFR) plots a one-dimensional time signal into a two-dimensional signal of frequency and time. TFR is widely used to analyze, synthesize, and modify non-stationary signal results in high accuracy, as both frequency and time are considered. STFT is the basic form of TFR, which generates narrow fragments of long-haul signals. Sufficiently narrow segments are observed as stationary and take the Fourier transform (FT) of every segment. Every FT shows the spectral details of the signal at a different time slice, which provides a simultaneous frequency and time estimation [31,32]. STFT of the EMG signal provides significant information about muscle activity during a task [22,32]. The generalized formula for STFT is

$$STFT_{x}\left(t,\omega \right)=\int_{-\infty }^{\infty }x\left(\tau \right)\omega \left(\tau -t\right)e^{-2\pi f\tau }d\tau ,$$

where x(τ) is the signal, ω(τ−t) is the observation window, and variable t slides the window over the signal, x(τ).

A spectrogram is the square magnitude of STFT that gives the power and energy distribution of the signal along the frequency direction at a specific time. For both spectrogram and STFT, there is a tradeoff between the frequency-based and time-based perspectives of a signal. The precision of time and frequency representation can be controlled by the size of the window chosen [33]. Spectrogram can be expressed as

$$STFT_{x}\left(t,\omega \right)={\left|\int_{-\infty }^{\infty }x\left(\tau \right)\omega \left(\tau -t\right)e^{-2\pi f\tau }\right|}^{2}d\tau ,$$

The spectrogram was drawn to see the frequency distribution of each signal. We used the Hanning window, as it has a low peak side slope, and it was suggested by Zawawi et al. in their EMG signal manual lifting study [33]. The presented frequency domain study may not specifically require the very short-time–frequency localization of the signal, which involves a narrow window size. Therefore, the size of the window was selected to be 1024, as it shows good visualization of the signal.

For the frequency domain signal analysis, the participant was asked to drive continuously for 1 h. The same driving conditions, electrode placements, and instructions as discussed in Section 2.3 were used. During the experiment, it was observed that remarkable changes in the driver’s behavior and EMG signal occur after half an hour of continuous driving. Thus, to analyze the frequency response of the resultant EMG signal, three samples were taken, the first at the start of the experiment, the second at the middle, and the third at 1 h of driving. As the frequency response of the EMG signal is more sensitive to movement artifacts. Thus, the 1-*min*-long samples were obtained to acquire more accurate information about muscle activity in the frequency domain. This experiment was repeated three times with one participant. 

### 2.7. Design Algorithm to Alert the Driver after Drowsiness Detection

A magnitude shift-based weak muscle activity detection algorithm was developed. The shift from alertness to drowsiness state causes a change in EMG signal magnitude, which can be detected using the developed algorithm. A 30 s-long test was conducted to assess the developed algorithm. The subject drives the simulated driving system for 30 s and deliberately changes the steering wheel grip. The steering wheel grip’s deliberate change was performed to replicate the real driving steering wheel grip control for active and drowsy driving. The subject first had a strong grip and then loosened the grip on the steering wheel; this was repeated three times to ensure reproducibility of the algorithm. One participant was tested for this study.

**Figure 4:**
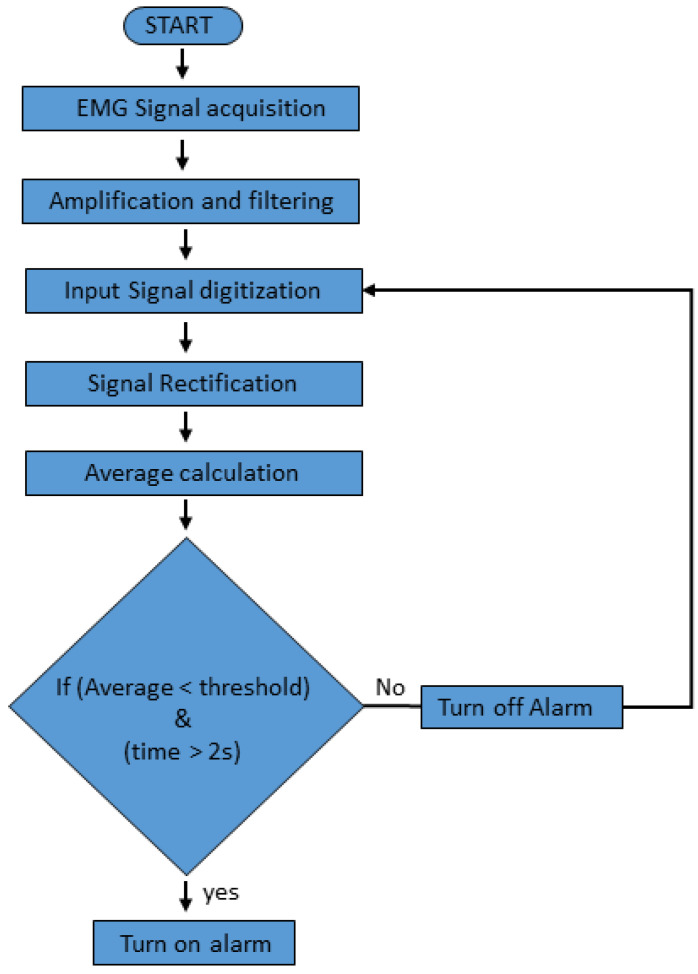
Flowchart of the proposed algorithm for driver drowsiness detection.

Figure 4 shows the flowchart of the developed algorithm. At first, the EMG signal was acquired from the driver’s forearm during driving using MNE. Secondly, amplification and filtering of the analog signal were performed. The signal was fed to the microcontroller for digitization. After digitization, the rectification of the EMG signal was conducted. The rectification of the EMG signal was performed by first turning all the negative values to positive and then taking the sum of all the values. Next, the average was calculated for every one second. By calculating the average, the power of the EMG signal can be summarized and represented by a single value for a selected time window. A threshold limit was set, and the average was monitored against this threshold. If the calculated average value crosses the threshold limit for a given time, the alarm will turn on. The time limit to alert the driver after drowsiness detection can be set accordingly.

We employed a traditional formula for calculating average, which is

$$Avg=\frac{\sum_{i=1}^{n}X_{i}}{n}$$

where n is the number of samples in one second, i.e., 100.

## 3. Results and Discussions

### 3.1. EMG Monitoring Performance of MNE vs. Ag/AgCl Electrode

**Figure 5:**
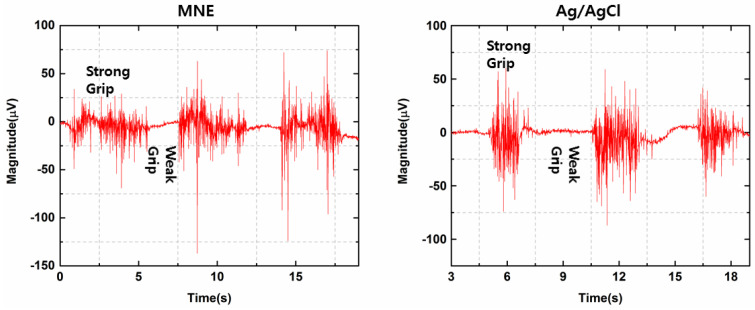
Comparison of MNE and Ag/AgCl electrode performance for EMG signal monitoring during strong and weak grip actions.

To assess the signal acquiring capability of MNE, the EMG signal was obtained using MNE and compared with the signal acquired by the Ag/AgCl electrode. Figure 5 shows the comparison of the EMG signal obtained using MNE and the Ag/AgCl electrodes. The amplitude of the signal during a strong grip was higher as compared with a weak grip, which is due to the potential difference generated due to the contraction and relaxation of the targeted muscles during grip action. The resultant EMG signal acquired with MNE is highly comparable with the signal acquired with the Ag/AgCl electrode. Hence, MNE is a good option for a minimally invasive EMG signal acquisition system; one full signal consists of strong and weak grip actions and the three repeated signals show the reproducibility of the experiment.

### 3.2. Driver Drowsiness vs. Muscle Activity Performance

Muscle activity and subjective drowsiness were analyzed for 10 min intervals whilst the subject was consecutively involved in the driving task for an hour. To analyze grip muscle activity, the average was calculated for the last 30 s of data from each 10 min interval. To analyze driver drowsiness level, the immediately reported score after every 10 min interval was used.

**Figure 6:**
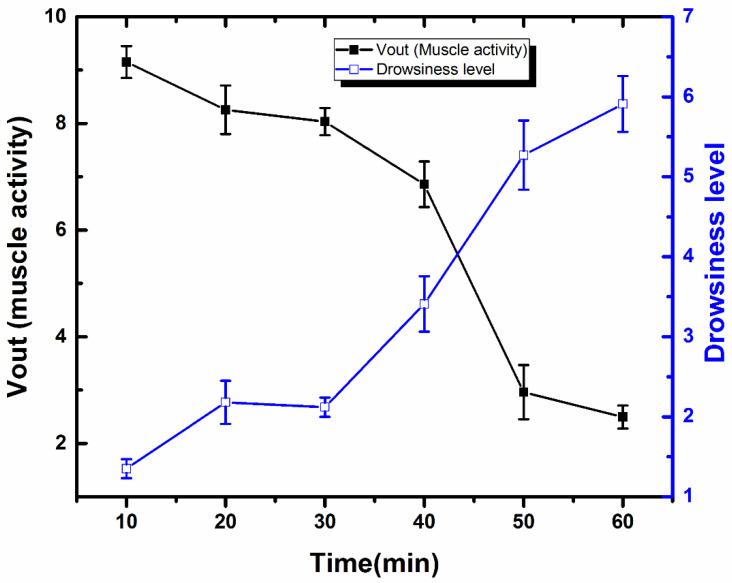
Degree of driver’s drowsiness and muscle activity over time. The data represents the average of three independent measurements with SE (n = 3).

The black line in Figure 6 shows the averages for muscle activity of steering wheel grip over time during interactive driving. The graph shows that muscle activity was high at the start of the driving task and tended to decrease over time. Specifically, at 10 min the average for the last 30 s was 9.15 (S.E = 0.3), at 20 min it was 8.25 (S.E = 0.45), 8.03 (S.E = 0.26) for 30 min, 6.85 (S.E = 0.42) for 40 min, 2.95 (S.E = 0.50) for 50 min, and 2.49 (S.E = 0.21) for 60 min. The data indicate that the subject’s muscle activity associated with steering wheel grip decreased slowly with time, and a high fall in the graph was recorded at 50 min of driving. The decrease in muscle activity over time is because of the increase in fatigue level.

In addition to muscle activity, the study also measured the participant’s drowsiness level while driving. The blue line in Figure 6 shows the trend in the variations in the degree of drowsiness. The level of the driver’s drowsiness increased over time in contrast with muscle activity, which decreased with time. In detail, at 10 min, the average was 1.35 (S.E = 0.1), at 20 min it was 2.18 (S.E = 0.27), 2.12 (S.E = 0.12) at 30 min, 3.41 (S.E = 0.34) at 40 min, 5.27 (S.E = 0.43) at 50 min, and 5.91 (S.E = 0.34) at 60 min. The participant was alert at the start of the task, so the measured drowsiness value was low. A small increase in drowsiness level was recorded at 10 min of the task, but there was a sudden increase in drowsiness level after 30 min of the driving task. Interestingly, a time difference between an increase in drowsiness level and a decrease in muscle activity was recorded; the drowsiness level increased after 30 min of the task, while the muscle activity decreased after 40 min. This might be because of the participant’s willingness to keep driving, which kept muscle activity high. The overall data show that the degree of driver drowsiness increased, while the muscle activity associated with steering wheel grip decreased over time. The results support the suggestion that the muscle activity data from steering wheel grip can be used to detect driver drowsiness levels.

### 3.3. Frequency Domain Response of the Signal

**Figure 7:**
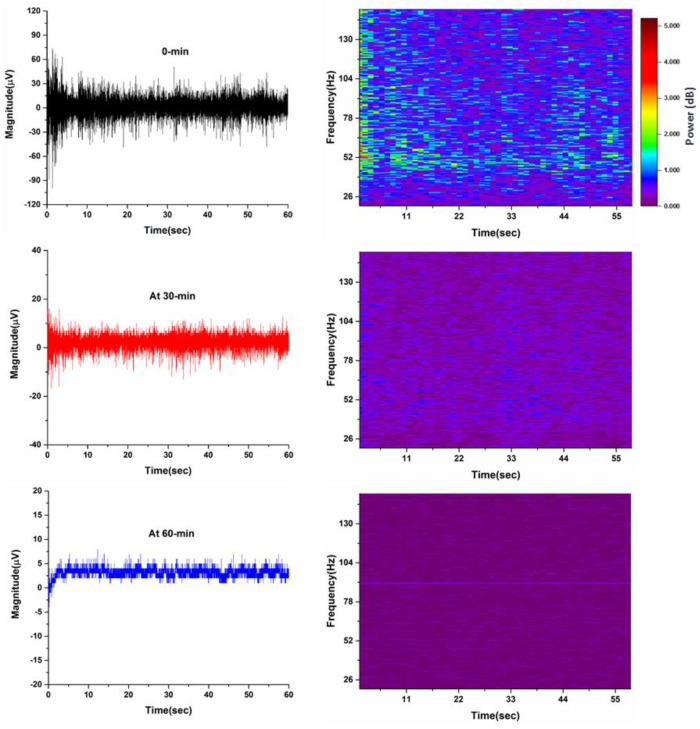
Raw EMG signals with different amplitudes at various times and their respective spectrograms. The color bar shows the magnitude of the frequency over time.

Figure 7 shows the three raw EMG signals and their respective spectrograms. The black raw signal shows the measured data at 0 min, the red raw signal represents the measured data at 30 min, and the blue raw signal represents the measured data at 60 min. The time-varying spectral representation of the signal is shown by the spectrogram. The spectrogram represents the time data in the *x*-axis, the frequency representation in the *y*-axis, and the amplitude of the frequency-time pair is color-coded.

In Figure 7, the high-frequency power contribution in the spectrogram of the EMG signal measured at 0 min of driving indicates high muscle activity due to the subject’s active driving and firm steering wheel grip. The spectrogram of the EMG signal measured at 30-min of driving shows low-frequency components, indicating decreased muscle activity due to an increase in the driver’s fatigue level, leading to drowsiness. The spectrogram of the third EMG signal taken after 1 h of driving represents minimal frequency components power, which indicates very low muscle activity due to the high drowsiness of the participant. The results indicate that the frequency components shift from the high-frequency spectrum to the low-frequency spectrum with an increase in drowsiness. The subject’s low-quality sleep before the experiment assisted in the conduct of the experiment.

### 3.4. Design Algorithm Performance for Drowsiness Detection and Alertness

**Figure 8:**
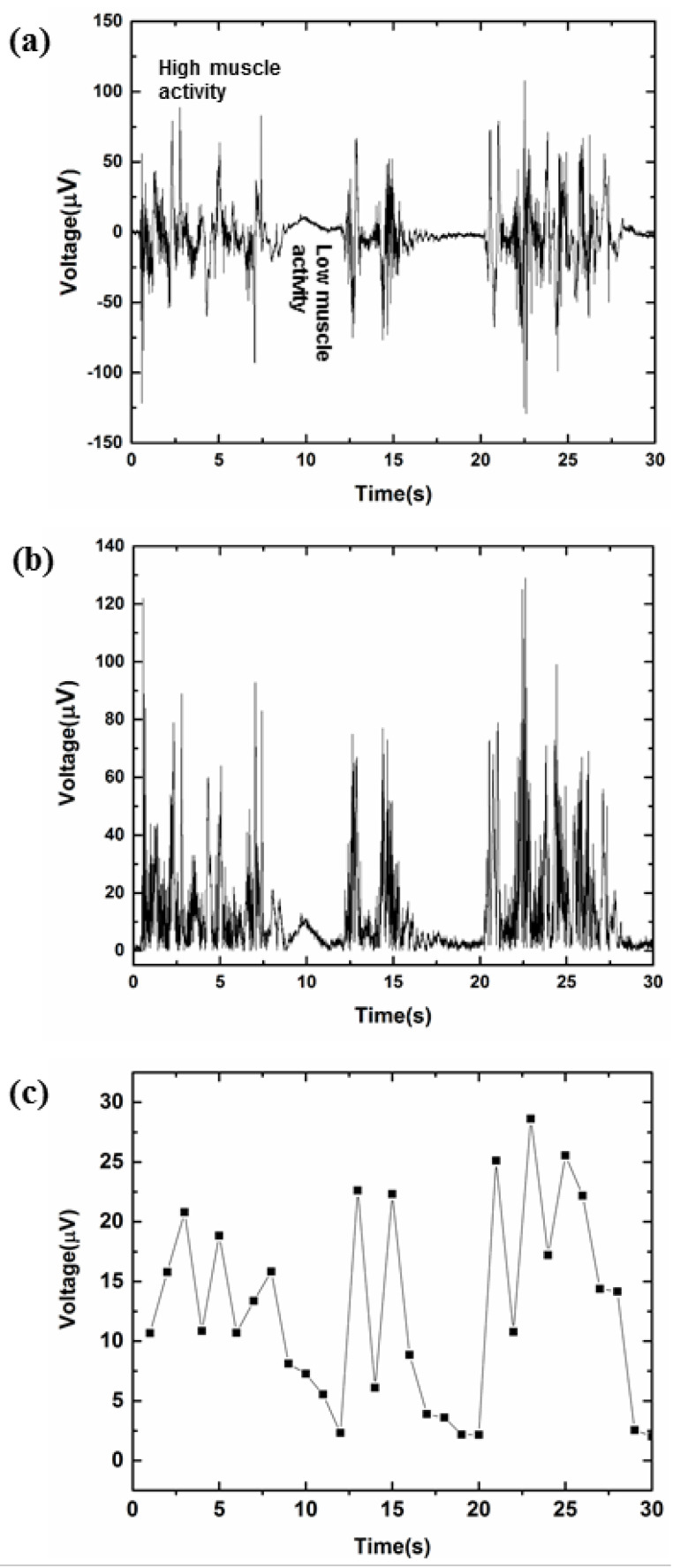
(**a**) Original signal obtained from forearm while driving; (**b**) Rectified EMG signal; (**c**) Average calculated every one second, while the dotted line represents the threshold set for driver drowsiness detection.

Figure 8a shows the voltage/time history of the EMG signal acquired from the forearm position by placing the electrodes on muscles involved in the steering wheel grip. The high amplitude signal represents high muscle activity due to the strong steering wheel grip, indicating active driving, while the low amplitude signal shows low muscle activity due to a weak steering wheel grip indicates an increase in the driver’s fatigue level, leading to drowsiness. Previous studies have also reported that during the transition from alertness to drowsiness, the EMG signal magnitude falls due to an increase in driver’s fatigue [34].

The one set of a complete signal consists of high muscle activity and low muscle activity with an increase and decrease in steering wheel grip during driving, while the three sets of repeated measurements presented in Figure 8a show the reproducibility of the test. Rectification of the raw EMG signal was performed before calculating the average. Figure 8b represents the rectified EMG signal. To detect low muscle activity, the average of the raw EMG signal was calculated and compared with the set threshold limit, as shown in Figure 8c. The dotted line in this figure shows the threshold limit set for detecting low muscle activity. It was observed that during active driving with a firm steering wheel grip by the participant, the average of the EMG signal did not fall below a certain value. Thus, the threshold limit can be set to this value. Further, because of the EMG signal variability between subjects, it is important to identify the threshold for an individual subject. Therefore, before experimenting with the developed algorithm, the driver’s active driving can be tested for a specific time to set the threshold limit. The resultant average signal was compared against the threshold limit, and the warning alarm system was turned on if the value fell below this limit. In this simulated driving experiment, we used a 3 second time interval for the detection of low muscle activity, while in real driving experiments, the detection time window could be different. The experimental results indicated that the proposed algorithm could detect the low muscle activity associated with steering wheel handgrip during driving, which leads to an increased degree of driver drowsiness. The proposed algorithm can detect and alert the drowsy driver in real time.

## 4. Conclusions

This study was conducted to identify the validity of detecting driver drowsiness by measuring the muscle activity associated with steering wheel grip. The change in muscle activity was measured while the participant was involved in simulated driving. Further, the participant’s drowsiness was measured to improve the validity of the measurements. STFT and spectrogram were used to examine the frequency domain features of the signal.

The results showed that the driver’s muscle activity of steering wheel grip decreased over time, while it decreased highly after 40 min of driving. In contrast, the driver’s drowsiness increased with time, and a high increase was observed after 30 min of driving. Therefore, as the drowsiness that the driver felt increased over time, the muscle activity associated with the handgrip decreased. Almost 10 minutes’ time difference was recorded between the increased drowsiness level and the decrease in steering wheel grip force. It could be said that the driver’s willingness to keep active induced the time difference between drowsiness and grip force. This time gap can help drivers to prepare for road safety before full sleepiness occurs. The results support the suggestion that a driver’s drowsiness can be detected from muscle activity associated with steering wheel grip.

The frequency-domain analysis of the signal showed that the frequency components move from a high-frequency spectrum to a lower frequency spectrum, with an increase in the driver’s fatigue level, which can further help in detecting a driver’s drowsiness. The muscle activity signal’s magnitude shift-based algorithm showed good performance for driver drowsiness detection and alertness. If the magnitude of the resultant signals drops below a certain level because of the low muscle activity caused by drowsiness, the algorithm detects the drop and alerts the driver. Based on the presented results, we propose some recommendations for future studies.

First, although the detection and alertness of a drowsy driver are possible based on muscle activity, as the EMG signal varies between individuals, the validity of the proposed algorithm needs to be more robustly tested with additional subjects and with different experimental designs. Second, the study was conducted in constant environmental conditions, such as room temperature and lighting. In real driving conditions, these and other environmental factors constantly change. Therefore, the performance of the proposed method should be further investigated in real driving conditions. Third, we suggest that future studies should examine the muscle activity signals from other body positions, such as the neck and shoulders, to investigate the effect of head movement during drowsiness. Fourth, a fusion of other physiological features such as EEG or EOG with muscle activity signals can improve the driver drowsiness detection method.

## References

[1] Ogilvie R.D., Wilkinson R.T., Allison S. (1989). The Detection of Sleep Onset: Behavioral, Physiological, and Subjective Convergence. Sleep.

[2] Soares S., Monteiro T., Lobo A., Couto A., Cunha L., Ferreira S. (2020). Analyzing Driver Drowsiness: From Causes to Effects. Sustainability.

[3] Lee M.L., Howard M.E., Horrey W.J., Liang Y., Anderson C., Shreeve M.S., O’Brien C.S., Czeisler C.A. (2016). High risk of near-crash driving events following night-shift work. Proc. Natl. Acad. Sci. USA.

[4] Bener A., Yildirim E., Özkan T., Lajunen T. (2017). Driver sleepiness, fatigue, careless behavior and risk of motor vehicle crash and injury: Population based case and control study. J. Traffic Transp. Eng..

[5] Watling C.N. (2020). Young drivers who continue to drive while sleepy: What are the associated sleep- and driving-related factors?. J. Sleep Res..

[6] Gwak J., Shino M., Hirao A. (2018). Early Detection of Driver Drowsiness Utilizing Machine Learning based on Physiological Signals, Behavioral Measures, and Driving Performance. Proceedings of the 2018 21st International Conference on Intelligent Transportation Systems (ITSC).

[7] Sahayadhas A., Sundaraj K., Murugappan M. (2012). Detecting Driver Drowsiness Based on Sensors: A Review. Sensors.

[8] Ingre M., Akerstedt T., Peters B., Anund A., Kecklucd G. (2006). Subjective sleepiness, simulated driving performance and blink duration: Examining individual differences. J. Sleep Res..

[9] Abdul Rahim H., Yusop Z., Abdul Rahim R. (2010). Drowsy Driver Detection via Steering Wheel. Sens. Transducers.

[10] Li F., Wang X.W., Lu B.L. (2013). Detection of driving fatigue based on grip force on steering wheel with wavelet transformation and support vector machine. Lect. Notes Comput. Sci..

[11] Baronti F., Lenzi F., Roncella R., Saletti R. (2009). Distributed sensor for steering wheel grip force measurement in driver fatigue detection. Proc.-Design Autom. Test Eur..

[12] Polychronopoulos A., Amditis A., Bekiaris E. (2004). Information data flow in awake multi-sensor driver monitoring system. Proceedings of the IEEE Intelligent Vehicles Symposium.

[13] Lin Y., Leng H., Yang G., Cai H. (2007). An Intelligent Noninvasive Sensor for Driver Pulse Wave Measurement. IEEE Sens. J..

[14] Awais M., Badruddin N., Drieberg M. (2017). A Hybrid Approach to Detect Driver Drowsiness Utilizing Physiological Signals to Improve System Performance and Wearability. Sensors.

[15] Hostens I., Ramon H. (2005). Assessment of muscle fatigue in low level monotonous task performance during car driving. J. Electromyogr. Kinesiol..

[16] Lin C.T., Wu R.C., Liang S.F., Chao W.H., Chen Y.J., Jung T.P. (2005). EEG-based drowsiness estimation for safety driving using independent component analysis. IEEE Trans. Biomed. Circuit Syst..

[17] Lin C.-T., Chang C.-J., Lin B.-S., Hung S.-H., Chao C.-F., Wang I.-J. (2010). A Real-Time Wireless Brain–Computer Interface System for Drowsiness Detection. IEEE Trans. Biomed. Circuits Syst..

[18] Chowdhury A., Shankaran R., Kavakli M., Haque M.M. (2018). Sensor Applications and Physiological Features in Drivers’ Drowsiness Detection: A Review. IEEE Sens. J..

[19] Dong-Mei H., Yi Y., Zheng W. (2009). Dong-Mei Measurement System for Surface Electromyogram and hand grip force based on Labview. World Congress on Medical Physics and Biomedical Engineering, Munich, Germany, 7–12 September 2009.

[20] Nazmi N., Abdul Rahman M., Yamamoto S.-I., Ahmad S., Zamzuri H., Mazlan S. (2016). A Review of Classification Techniques of EMG Signals during Isotonic and Isometric Contractions. Sensors.

[21] Sidek S.N., Haja Mohideen A.J. (2012). Mapping of EMG signal to hand grip force at varying wrist angles. Proceedings of the 2012 IEEE-EMBS Conference on Biomedical Engineering and Sciences.

[22] Dantas J.L., Camata T.V., Brunetto M.A.O.C., Moraes A.C., Abrao T., Altimari L.R. (2010). Fourier and wavelet spectral analysis of EMG signals in isometric and dynamic maximal effort exercise. Proceedings of the 2010 Annual International Conference of the IEEE Engineering in Medicine and Biology.

[23] Satti A.T., Park J., Park J., Kim H., Cho S. (2020). Fabrication of Parylene-Coated Microneedle Array Electrode for Wearable ECG Device. Sensors.

[24] Procházka A., Kuchyňka J., Vyšata O., Cejnar P., Vališ M., Mařík V. (2018). Multi-Class Sleep Stage Analysis and Adaptive Pattern Recognition. Appl. Sci..

[25] Analog Devices, Heart Rate Monitor for Wearable Products, Massachusetts, USA, 2012–2017. https://www.analog.com/media/en/technical-documentation/data-sheets/ad8232.pdf.

[26] De Luca C.J., Donald Gilmore L., Kuznetsov M., Roy S.H. (2010). Filtering the surface EMG signal: Movement artifact and baseline noise contamination. J. Biomech..

[27] Roland T., Amsuess S., Russold M., Baumgartner W. (2019). Ultra-Low-Power Digital Filtering for Insulated EMG Sensing. Sensors.

[28] (2017). AD8232 Filter Design (Open Source). https://www.analog.com/en/products/ad8232.html#product-tools.

[29] Horne J., Reyner L. (1999). Vehicle accidents related to sleep: A review. Occup. Environ. Med..

[30] Blanc Y., Ugo D. (2010). Electrode Placement in Surface Electromyography(sEMG) “Minimal Crosstalk Area”. Open Rehabil. J..

[31] Shair E.F., Ahmad S.A., Marhaban M.H., Mohd Tamrin S.B., Abdullah A.R. (2017). EMG Processing Based Measures of Fatigue Assessment during Manual Lifting. Biomed Res. Int..

[32] Costa M.V., Pereira L.A., Oliveira R.S., Pedro R.E., Camata T.V., Abrão T., Brunetto M.A.O.C., Altimari L.R. (2010). Fourier and wavelet spectral analysis of EMG signals in maximal constant load dynamic exercise. IEEE Eng. Med. Biol. Soc. Conf..

[33] Zawawi T.N.S.T., Abdullah A.R., Halim I., Shair E.F., Salleh S.M. (2016). Application of spectrogram in analysing electromyography (EMG) signals of manual lifting. ARPN J. Eng. Appl. Sci..

[34] Mahmoodi M., Nahvi A. (2019). Driver drowsiness detection based on classification of surface electromyography features in a driving simulator. Proc. Inst. Mech. Eng. Part H J. Eng. Med..

